# Quality of life and its predicting factors for Tunisian children with cerebral palsy

**DOI:** 10.4102/ajod.v11i0.1046

**Published:** 2022-12-15

**Authors:** Ghanmi Marwa, Sahbi Mtawaa, Emna Toulgui, Rihab Moncer, Walid Wannes, Khaled Maaref, Sonia Jemni

**Affiliations:** 1 Department of Physical Medicine and Rehabilitation, Faculty of Medicine of Monastir, University Hospital Sahlou, Sousse, Tunisia; 2 Department of Physical Medicine and Rehabilitation, Faculty of Medicine of Sousse, University Hospital of Kairouan, Kairouan, Tunisia; 3 Department of Physical Medicine and Rehabilitation, Faculty of Medicine of Sousse, University Hospital of Sahloul, Sousse, Tunisia

**Keywords:** cerebral palsy, quality of life, CP QOL-Child questionnaire, child, ICF

## Abstract

**Background:**

Cerebral palsy (CP) can cause motor, sensory, perceptual, cognitive, communication and behavioural disorders. The complexity of this condition justifies measuring the quality of life (QOL) of children with CP. This measurement depends on personal and socio-economic factors, hence the relevance of performing it in our cultural context of Tunisia.

**Objectives:**

The objectives of this study were to assess the QOL of Tunisian children with CP and to identify predictive factors for QOL.

**Method:**

A cross-sectional study using a self-administered questionnaire (the CP QOL-Child) was employed. It included 68 children with CP and their parents who consulted the outpatient clinics of Physical Medicine and Rehabilitation of the University Hospital of Sahloul Sousse.

**Results:**

The QOL of children with CP was altered, and the mean total score for the CP QOL-Child was 59.3 (± 14). All domains were affected by this alteration. Six predictive factors for lowered QOL in children with CP were identified, namely age older than 6 years, swallowing disorders, more intense chronic pain, greater level of motor impairment, the use of botulinum toxin injection and the absence of verbal communication.

**Conclusion:**

Intervention with children with CP must be mindful of their altered QOL. Five out of the six predictive factors of QOL are modifiable through a multidisciplinary approach within the framework of the International Classification of Functioning, Disability and Health (ICF).

**Contribution:**

The multiplicity of the factors associated with QOL revealed by this study incites clinicians to adopt the ICF approach by displaying its practical implications on the efficiency of the medical intervention.

## Introduction

Cerebral palsy (CP) refers to a group of pathologies secondary to a non-progressive injury of the developing central nervous system occurring in a child under three years of age. This injury can cause motor disorders often associated with sensory, perceptive, cognitive, communication and behaviour disorders, epilepsy and secondary musculoskeletal problems (Rosenbaum et al. 2007). It is the most common cause of disability in children in developed countries with a prevalence range from 1.4 to 2.1/1000 live births (Galea et al. 2019).

Results from developing countries are divergent but an overall higher prevalence ranging from 3.4 to 4.1/1000 live births is reported (Gladstone 2010; Serdarog 2006). Data concerning prevalence of CP in Tunisia is unknown because of the lack of a dedicated register. Cerebral palsy can cause functional deficits and the inability to perform daily-life activities, which, as a result, compromise functional independence, participation in social life and quality of life (QOL). In 2006, Varni conducted a study comparing QOL measured by a generic self-administered questionnaire, in children diagnosed with multiple chronic conditions (10 chronic conditions, 33 categories according to severity) and children in good apparent health. The most altered QOL was found in children with CP (Varni et al. 2006).

The International Classification of Functioning, Disability and Health (ICF) offers a more comprehensive and standardised approach to study the impact of disabling diseases, such as CP, on functioning (Meucci et al. 2014) by integrating the bio-psycho-social dimensions into the management strategy. This classification has also identified the concept of QOL as the main objective of all therapeutic interventions. The study of QOL of patients has thus gradually become an important measure to evaluate the effectiveness of treatments for children with CP (Maenner et al. 2016).

As defined by the World Health Organization (WHO), QOL is the individual’s perception of their place in life, in the context of their cultural and value system and in relation to their proper goals and concerns (Kuyken et al. 1995). Therefore, the measurement of QOL must include the opinions and perceptions of patients and their families. Then, it must be based on the use of standardised and validated questionnaires. Among the most used, are the Cerebral Palsy Quality of Life Questionnaire for Children (CP -QOL-Child) (Waters et al. 2009), the Child Health Questionnaire (CHQ) (Schneider et al. 2007) and the European generic health-related QOL questionnaire (KIDSCREEN) (Ravens-Sieberer et al. 2005). Others are less used, such as the Paediatric Quality of Life Inventory (Varni et al. 2006) and the Caregiver Priorities and Child Health Index of Life with Disabilities (Narayanan et al. 2006). Cerebral palsy Quality of Life-Child was selected by a systematic review of the literature published in 2014 as the most appropriate measurement instrument that takes into account all the characteristics of CP while representing the concepts of the ICF (Schiariti et al. 2014). The strength of CP QOL-Child is shown through its design, which took into account the perceptions and experiences of children with CP and their families (Waters et al. 2006). Indeed, many researchers consider the CP QOL-Child to be the gold standard for this evaluation (Davis et al. 2010). The CP QOL-Child was validated through extensive research with good test–retest reliability, construct validity and internal consistency (Waters et al. 2006). This instrument was also translated and validated in Arabic with excellent test–retest reliability, good internal consistency, an intraclass coefficient between 0.88 and 0.97 and a Cronbach’s alpha coefficient, which exceeds 0.7 (El-Weshahi et al. 2017). The starting age of 4 years was chosen because it is the ideal age for CP diagnosis. Children over 12 years old were not included as it is possible that new issues, such as body image, pressure from school and employment, will arise during adolescence.

The main objective of this study is to assess the QOL of children with CP by the CP QOL-Child questionnaire and to determine predictive factors for lowered QOL.

## Research methods and design

### Study design

This study employed a cross-sectional design commonly used to investigate associations between risk factors and the outcome that is QOL. This design is useful for public health issues such as CP and for the generation of hypotheses but it is limited in time (Wang & Cheng 2020).

### Setting

Data collection took place in Physical Medicine and Rehabilitation outpatient clinics of the University Hospital of Sahloul, Sousse between 01 September 2019 and 31 March 2020. Parents taking care of these children were also included. There are only six outpatient clinics for Physical Medicine and Rehabilitation in Tunisia. Those of the University Hospital of Sahloul receive patients from all over the centre and the east region of the country.

### Study population

All the children with CP, aged from 4 to 12 years old and their parents were included. However, those who were also diagnosed with genetic syndromes, heart disease, diabetes or cancer and those with acute traumatic or infectious disease were excluded. Also, parents who refused to answer the questionnaire and those who did not have verbal or written skills in Arabic to complete the measuring instruments and the consent form were excluded.

Among the 123 children who presented with CP and their parents during the data collection period, of which 68 were included in this study.

### Data collection

Within the framework of the ICF, a trained physiatrist collected information about age, gender, patient history (comorbidities, medical and surgical neuro-orthopaedic interventions), socioeconomic status (household area, the parents’ level of education, the parents’ profession, monthly income, health insurance, the number of siblings, if they had a sibling with disability), type of school the child attends (kindergarten, mainstream school, school for learners with special educational needs).

Then, he conducted a physical examination including a neurological, neurosensory and neuromotor assessment, an algo-functional assessment by the visual analogue scale (VAS) and the Gross Motor Function Scale (GMFCS) and a psychological assessment using self-administered questionnaires (Hariz et al. 2013; Pashmdarfard et al. 2017; Suleiman, Hadid & Duhni 2012):

- the Child Depression Rating Score (CDRS)
- the Screen for Child Anxiety Related Disorder (SCARED) in its Arabic version for anxiety and
- the Pittsburg Sleep Quality Index (PSQI) in its Arabic version for the subjective assessment of sleep quality.

Quality of life was measured using the CP QOL-Child. It assesses seven areas of QOL, namely ‘social well-being and acceptance’ (SWA); ‘feelings about functioning’ (FAF); ‘participation and physical health’ (PHP); ‘emotional well-being and self-esteem’ (EWS); ‘access to services’ (AS); ‘pain and impact of disability’ (PID) and ‘family health’ (FH).

Cerebral palsy Quality of Life-Child is made of two versions to be used together when possible: the proxy reported version containing 66 items, and the self-administered version containing 52 items. The self-administered version was used according to the manual of instructions in children with CP aged between 9 and 12 years old and having preserved intellectual level and verbal communication. Where the use of both versions was possible, the scores of the two versions for each domain were averaged and used in the analytical study (Waters, Boyd & Reddihough 2013).

### Definitions of variables

- **Monthly household income:** A monthly household income of less than 1000 Tunisian Dinars was considered low, moderate when between 1000 and 1400 Dinars and good when above 1400 Dinars.
- **Intensity of chronic pain**: In accordance with the recommendations of the High Authority for Health, the patient’s pain was considered weak for a VAS between one and three, moderate when it is between four and five, intense between six and seven and unbearable for a VAS greater than or equal to eight.
- **Anxiety disorder:** It was diagnosed for a SCARED score > 25.
- **Clinical depression:** It was defined by a CDRS score Greater than or equal to 30.
- **Sleep disorder:** It was retained with a PSQI score > 5.
- **Motor function impairment**: It was defined functionally by the GMFCS classification system.
- **Quality of life scoring**: The response to each question was converted into a percentage varying from 0 to 100 according to the coding algorithm (Waters et al. 2013). One hundred corresponding to the highest possible QOL according to the child with CP and or the parent included. Then, the average of the percentages of the predefined responses for each domain was calculated. Seven averages of percentages varying from 0 to 100 were thus obtained. The CP QOL-Child total score (CP-QOL total) is the average of the seven domains. The higher the CP-QOL total, the better the QOL.

### Data analysis

The collected data were saved and analysed using SPSS version 22 ‘Statistical Package for Social Sciences’ software. Qualitative variables were described by counts and percentages. Quantitative variables with normal distribution were described as means and standard deviations, those with non-normal distribution were described as medians with interquartile ranges. The association between QOL and 44 elements the ICF was analysed. Comparisons of two means were made using Student’s *t*-test for independent samples. Comparisons of multiple means were made using the ANOVA test. To determine the predictive factors of CP QOL-Child score, a multiple linear regression was performed. The normal distribution of this score was checked graphically beforehand. When the linearity between the quantitative variables and the CP QOL-Child score was not applicable, they were transformed into qualitative variables. Factors that were associated with changes in CP QOL-Child scores with a *p* < 0.20 were included in the model. After obtaining the final model, the conditions of normality, linearity and homoscedasticity of the residuals were verified. A significance level of 5% was set for all statistical tests performed.

### Ethical considerations

This study design was approved by the Committee of Ethics of the Faculty of Medicine of Monastir Tunisia. We obtained written consent of the author of the validated Arabic version of the CP-QOL questionnaire. Also, free and informed written consent for participation and publication of the parents accompanying the child with CP was obtained after using forms detailing the purpose and modalities of participation. We ensured confidentiality by anonymous coding of the files that were kept in a specific binder. The study design was approved by the Committee of Ethics of the Faculty of Medicine of Monastir Tunisia.

## Results

### Descriptive study

The parent caring for the child with CP who responded to our questionnaire was the mother 97.1% of the time. The age of the children varied between 4 and 12 years with a mean age of 7.99 (± 2.82). Gender ratio was 0.83. A total of 98.5% of the parents were living together. Only 4.4% of the included children with CP had no siblings. Mean siblings’ number was 2.12. Twenty-three children with CP (33.8%) had a brother or sister with disability. The VAS score ranged from 0 to 9 with a median of 0 and an interquartile range of 0–2.25. Seven children with CP or 9.8% of our population, had a SCARED score > 25, which indicates the presence of anxiety. The assessment of the quality of sleep in children with CP showed an average PSQI value of 6.75 (± 6.04). A total of 37.9% of the children with CP presented with sleep disturbance (PSQI > 5). A CDRS score of > 30 was indicative of clinical depression in 4.41% of the children with CP.

A total of 51 participating children with CP (75%) benefited from regular rehabilitation with a frequency of three sessions per week in 61.8% of cases. Difficulty in accessing functional rehabilitation was reported by 29.4% of participating parents. Only 8 children (11.8%) received speech therapy (Table 1).

**TABLE 1:** Biographical information of children with cerebral palsy and their families (*N* = 68).

Demographic and clinical characteristics	*n*	%
**Biographic characteristics**		
**Mother’s profession**		
Public sector employee	17	25.0
Private sector employee	9	13.2
Housewife	42	61.8
**Mother’s education**		
None	10	14.7
Primary	22	32.4
Secondary	17	25.0
University	19	27.9
**Father’s profession**		
Public sector employee	9	13.2
Private sector employee	16	23.5
Free work	42	61.8
Unemployment	1	1.5
**Father’s education**		
None	7	10.3
Primary	28	41.2
Secondary	13	19.1
University	20	29.4
**Household area**		
Urban	42	61.8
Rural	26	38.2
**Health isurance**		
Insurance	50	73.5
Social help	18	26.5
**Monthly household income**		
Low	38	55.9
Moderate	24	35.3
Good	6	8.8
**Clinical characteristics of children with CP**		
**Topographic form**		
Diplegia	13	19.1
Hemiplegia	15	22.1
Quadriplegia	40	58.8
Triplegia	0	0
**Neurological symptoms**		
Hypotonia	4	5.9
Spasticity	63	92.6
Dystonia	21	30.9
Ataxia	12	17.6
**Associated disorders**		
Epilepsy	17	32.1
Autism	5	9.4
Intellectual deficit	31	45.5
Language disorders	36	52.9
Digestive disorders	37	54.5
Swallowing disorders	24	45.3
Drooling	22	41.5
Vesico-sphincteric disorders	32	47.1
Respiratory disorders	5	7.4
**Hearing impairment**		
Hypoacusis	5	7.3
Deafness	1	1.5
**Visual disturbances**		
Blindness	1	1.5
Strabismus	7	10.3
Amblyopia	7	10.3
Agnosia	2	2.9
Anophthalmos	1	1.5
**Treatment informations**		
**Orthopaedic devices**		
Cruro-pedal posture splint	29	42.6
Corset seat	8	11.7
Corset	6	8.8
Wheelchair	21	30.9
Tibial foot splint	9	13.2
**Surgical treatment**		
Achilles tendon lengthening	8	11.7
Twin blade lengthening	3	4.4
Tenotomy of adductors	5	7.3
Hip dislocation surgery	3	4.4
**Botulinum toxin injection**		
Yes	41	60.3
No	27	39.7

CP, cerebral palsy.

The mean total CP QOL-Child score (CP-QOL total) was 59.3 (± 14.0). The means of the various domains measured are detailed in Table 2. The mean confidence of mothers in understanding their children’s feelings was 75.2 (± 21.2) (Table 2).

**TABLE 2:** Results of the cerebral palsy quality of life-child calculated in children with cerebral palsy.

QOL scores measured by the CP QOL-child	Mean	Standard deviation	Confidence interval
Child’s CP-QOL total score	59.3	14.0	55.9; 62.7
Social well-being and acceptance	73.9	16.4	70.0; 77.9
Participation and physical health	54.6	23.5	48.9; 60.3
Feelings about functioning	59.2	24.7	53.3; 65.2
Emotional well-being and self-esteem	62.8	15.4	57.1; 64.5
Access to services	57.2	19.0	52.6; 61.8
Pain and feelings of disability	39.1	17.3	34.9; 43.3
Family health	48.2	22.4	42.8; 53.7
Confidence of mothers in understanding children’s feelings	75.2	21.2	70.0; 80.3

CP, cerebral palsy; QOL, quality of life.

Both versions of CP QOL-Child could be used in 10.3% of children.

No statistically significant differences were found when comparing the results of the CP-QOL child calculated from the self-administered and the proxy-reported versions Table 3.

**TABLE 3:** Comparison of the results of the cerebral palsy quality of life-child calculated from the two versions in children with cerebral palsy aged over 9 years with normal intellectual capacities and possible verbal communication.

CP QOL-child scores	Parents version		Children version		*p*-value
	Mean	s.d	Mean	s.d	
CP-QOL total	52.9	11.4	62.3	4.7	0.974
Social well-being and acceptance	66.5	11.9	66.3	6.95	0.070
Feelings about the functionning	48.4	20.5	65.5	3.8	0.111
Participation and physical health	47.9	24.7	65.5	3.9	0.398
Emotional well-being and self-esteem	58.1	18.4	65.1	7.6	0.602
Pain and feelings of disability	46.9	16.7	42.6	13.3	0.066

CP, cerebral palsy; QOL, quality of life; s.d., standard deviation.

### Analytical study

#### Univariate study of the quality of life of children with cerebral palsy

Fathers working in the public sector had better CP-QOL total scores for their children (*p* = 0.042). In addition, the mean CP-QOL total was significantly higher in children integrated in kindergartens, mainstream schools or schools for learners with special educational needs compared with non-integrated children (*p* < 0.001).

Children with an intellectual disability had a significantly more altered QOL (*p* = 0.006). Cerebral palsy-Quality of Life total values were significantly higher in children with verbal communication (*p* ≤ 0.001). Children with CP who drool had significantly lower QOL than in absence of drooling (*p* ≤ 0.001). In contrast, the presence of swallowing disorders was not associated with a difference in CP QOL-Child scores (*p* ≤ 0.102). Cerebral palsy Quality of Life-Child scores in children with CP with vesico-sphincteric disorders were significantly lower than in the absence of these disorders (*p* ≤ 0.001).

The CP-QOL total was significantly higher in the hemiplegic form than in the diplegic form. The quadriplegic form of CP was associated with the most altered QOL (*p* < 0.001). Likewise, a significant difference between the CP-QOL total scores according to the GMFCS classification levels was found. The higher the GMFCS, the lower the total score of the CP-QOL questionnaire, indicating a more impaired QOL (*p* < 0.001). The intensity of chronic pain was also associated with a significant difference in QOL. In fact, the more intense the pain, the lower the scores, that is, the more the QOL is altered (*p* = 0.005). However, the spastic form was not associated with an alteration in QOL (*p* = 0.485). Furthermore, CP-QOL scores were significantly lower in children with CP who have sleeping disorders as revealed by the PSQI score (*p* = 0.042). Finally, no significant difference between CP-QOL total scores in children with CP who underwent different kinds of therapeutic interventions was found (Table 4).

**TABLE 4:** Relationship between quality of life in children with cerebral palsy and socio-demographic, economic, clinical and therapeutic information.

Biographical information	CP-QOL total		*p*-value
	Mean	s.d	
**Age**			0.103
< 6 years	64.1	11.0	-
> 6 years	57.7	14.8	-
**Gender**			0.440
Male	60.7	13.6	-
Female	58.1	14.7	-
**Number of siblings**			0.281
< 2	58.2	14.9	-
> 2	62.4	11.8	-
**Sibling with disability**			0.663
Yes	60.3	15.5	-
No	58.7	13.5	-
**Father’s education**			0.817
None or primary	59.7	12.9	-
Secondary or more	58.9	15.3	-
**Mother’s education**			0.080
None or primary	62.2	13.0	-
Secondary or more	56.2	14.8	-
**Mother’s occupation**			0.360
Civil servant	57.3	11.9	-
Housewife	60.5	15.4	-
**Father’s occupation**			0.042*
Private sector employee	57.3	14.3	-
Public sector employee	65.3	12.3	-
**Social level**			0.655
Average or good	60.2	14.7	-
Low	58.6	13.9	-
**Household area**			0.583
Urban	58.5	15.2	-
Rural	60.5	12.6	-
**Social cover**			0.225
Low income	62.8	12.9	-
Good	58.0	14.5	-
**School integration**			< 0.001*
Yes	67.3	10.4	-
No	51.7	14.0	-
**Epilepsy**			0.712
Yes	58.2	12.4	-
No	59.7	14.8	-
**Autism**			0.122
Yes	49.8	21.5	-
No	60.0	13.4	-
**Intellectual deficit**			0.006*
Yes	63.5	13.8	-
No	54.2	13.1	-
**Communication**			< 0.001*
Verbal	68.0	10.6	-
Non-verbal	51.5	12.3	-
**Drooling**			0.007*
Yes	53.8	13.0	-
No	63.1	13.8	-
**Swallowing disorders**			0.102
Yes	55.5	15.6	-
No	61.3	13.1	-
**Urinary dysfunction**			< 0.001*
Yes	66.5	11.9	-
No	51.3	12.4	-
**Digestive disorders**			0.579
Yes	60.1	12.5	-
No	58.3	16.0	-
**Hearing impairment**			0.710
Yes	59.3	13.7	-
No	59.3	15.8	-
**Vision impairment**			0.971
Yes	59.5	14.5	-
No	57.3	10.5	-
**Topographic form**			< 0.001*
Hemiplegia	73.2	11.1	-
Diplegia	60.3	16.4	-
Quadriplegia	53.7	10.5	-
**GMFCS**			< 0.001*
I	78.9	7.5	-
II	69.5	8.5	-
III	63.5	9.5	-
IV	63.5	8.1	-
V	51.6	13.0	-
**Chronic pain**			0.005*
Absent	62.7	11.8	-
Low	60.5	13.5	-
Average	37.7	7.3	-
Intense	52.0	19.9	-
Insufferable	52.5	16.2	-
**Spasticity**			0.485
Yes	59.9	13.4	-
No	52.1	22.2	-
**Anxiety**			0.065*
Yes	46.7	10.2	-
No	60.0	14.0	-
**Depression**			0.937
Yes	59.9	19.2	-
No	59.3	14.1	-
**Sleep disturbance**			0.042*
Yes	54.8	15.8	-
No	62.3	12.3	-
**Apparatus**			0.455
Yes	62.5	10.2	-
No	59.9	13.5	-
**Functional rehabilitation**			0.128
Yes	63.8	13.1	-
No	57.8	14.3	-
**Speech therapy**			0.439
Yes	55.8	14.0	-
No	59.8	15.5	-
**Botulinum toxin**			0.107
Yes	57.2	15.6	-
No	62.5	11.1	-
**Hip dislocation surgery**			0.149
Yes	50.2	9.8	-
No	61.3	13.0	-

CP, cerebral palsy; QOL, quality of life; GMFCS, Gross Motor Function Scale; s.d., standard deviation.

* , *p* < 0.05.

#### Multivariate analysis

Factors integrated in the initial model of multiple linear regression were 18. The most influencing factors on the QOL of children with CP identified by our analyses were age over 6 years, the presence of swallowing disorders, the level of the motor impairment assessed by the GMFCS, the intensity of chronic pain and the use of botulinum toxin, which had a negative influence on the total CP-QOL score. In contrast, children with CP with sustained verbal communication had better QOL than those with little or no functional speech (Table 5).

**TABLE 5:** The predictive factors of quality of life of children with cerebral palsy objectified by cerebral palsy-quality of life total.

Predictive factors	Α	CI	Β	*p-*value
Age > 6 years	−6.1	(-11.2; -1.0)	−0.188	0.019*
Oral communication	10.1	(5.1; 15.1)	0.360	< 0.001*
Swallowing disorders	−6.6	(-11.9; -1.3)	−0.224	0.015*
GMFCS	−2.4	(-4.3; -0.4)	−0.200	0.019*
Chronic pain	−5.1	(-6.9; -3.3)	−0.518	< 0.001*
Botulinum toxin	−6.6	(-11.1; -2.0)	−0.229	0.005*

CP, cerebral palsy; QOL, quality of life; GMFCS, Gross Motor Function Scale; α, non-standardised coefficient; CI, confidence interval; β, standardised coefficient; *p*, risk of error.

* , *p* < 0.05.

## Discussion

The mean score of the CP-QOL child in this study was lower than those found by most of the studies carried out in developed countries (Davis et al. 2010), but also in developing countries (Angreany et al. 2015; Atasavun Uysal et al. 2016; Braccialli et al. 2016; Power et al. 2018; Soleimani et al. 2015). Only one study using the Arabic version of the CP QOL-Child, which was carried out in Egypt, found lower values than in the study at hand (El-Weshahi et al. 2017) (Table 6).

**TABLE 6:** Comparative table of the means of total score of cerebral palsy quality of life questionnaire between the different studies in the literature.

Study	Population	Country	Mean CP-QOL total
Angreany (Angreany et al. 2015)	60	Indonesia	63.7
Atasavunu (Atasavun Uysal et al. 2016)	149	Turkia	63.5
Soleimani (Soleimani et al. 2015)	200	Iran	60.8
DavisE (Davis et al. 2010)	204	Australia	62.7
Braccialli (Braccialli et al. 2016)	113	Brazil	69.5
El-Weshahi (El-Weshahi et al. 2017)	200	Egypt	41.0
This study†	68	Tunisia	59.3

CP, cerebral palsy; QOL, quality of life.

† , s.d. = 55.9; 62.7.

This divergence could be explained by the sociocultural differences and the difference between the characteristics of the populations studied and the therapeutic behaviours. In fact, the predominant topographic distribution in our population was quadriplegia (58.8%), followed by hemiplegia (22%).

All areas of QOL are affected by CP. The best score was observed for the area of ‘Social welfare and acceptance’ (73 CI = [70.0; 78.0]). The area with the lowest score was the ‘Pain and feelings of disability’ (39 CI = [34.9; 43.3]). These same results were found by the various published studies. Also, CP affects the whole family as indicated in this study’s results with the low score around ‘Family health’ compared with other areas. Indeed, several studies that have evaluated the burden of this condition on caregivers have concluded that parents experience a difficult ordeal and see their mental, physical and psychological state affected by the presence of a disabled child in the family (Ben Salah Frih et al. 2010; Brehaut et al. 2004; Davis et al. 2010) (Table 7).

**TABLE 7:** Comparative tables of the means found in the literature in each of the domains of the cerebral palsy quality of life-child.

StudyCP-QOL domain	Angreany (Angreany et al. 2015)	Atasavunuan (Atasavun Uysal et al. 2016)	Soleimani (Soleimani et al. 2015)	Davis E (Davis et al. 2010)	Brachialli M (Braccialli et al. 2016)	Heba MT (El-Weshahi et al. 2017)	This study
SWA	67.4	73.7	74.1	73.0	80.8	53.7	73.9
FAF	55.8	69.0	63.3	64.1	70.8	37.5	54.6
PHP	62.6	65.8	66.3	65.4	75.0	39.7	59.2
EWSE	62.1	74.4	70.5	70.4	85.4	36.8	60.8
AS	68.0	50.1	56.1	55.6	75.0	38.3	57.2
PFD	53.0	40.3	46.5	44.6	31.3	40.1	39.1
FH	77.0	71.2	48.5	50.7	75.0	41.1	48.2

Note: Please see the full reference list of the article, Marwa, G., Mtawaa, S., Toulgui, E., Moncer, R., Wannes, W., Maaref, K. et al., 2022, ‘Quality of life and its predicting factors for Tunisian children with cerebral palsy’, *African Journal of Disability* 11(0), a1046. https://doi.org/10.4102/ajod.v11i0.1046, for more information.

SWA, social welfare and acceptance; FAF, feelings about functioning; PHP, participation and physical health; EWSE, emotional well-being self-esteem; AS, access to services; PFD, pain and feeling of disability; FH, family health.

In children with CP for whom both versions could be used, the self-administered version completed by the child and the proxy-reported version completed by the parents, comparable results in the different domains, as well as for the CP-QOL total were found. These results were in agreement with the results of Waters et al. (2006). Thus, it can be concluded that in the presence of difficulties preventing the child from completing the questionnaire themselves, the parents’ responses offer a good estimate of the children’s QOL (Waters et al. 2006). It should also be observed that several studies using other scores found that children with CP report better QOL than reported by their parents because of the added anxiety and stress of the parent (Makris, Dorstyn & Crettenden 2019), hence the relevance of using both versions when possible, and the importance of completing the QOL assessment by referring to other stakeholders such as siblings, teachers, school friends and the healthcare team.

Ten factors were associated with QOL in children with CP. The identified factors can be classified according to the ICF in deficiencies of the organic functions (intellectual or speech deficit, drooling, vesico-sphincteric disorders, the intensity of the pain and the topographic form of the injury), limitation of activities (GMFCS score), restriction of participation (school integration), personal factors (sleep disorders) and environmental factors (father’s profession). These results encourage clinicians to investigate further all aspects of the bio-psycho-social model for they have a quantifiable repercussion on QOL. The multiplicity of these factors reflects the complexity of the initial assessment and the need for multidisciplinary management.

Six predictive factors for QOL were found, namely age, verbal communication, intellectual disability, motor impairment, pain intensity and the use of botulinum toxin. This model did not contain socio-demographic and economic factors, which is in line with the results found in 2017 by Rappas part of the SPARCLE study (Thorley et al. 2012) where a longitudinal study of QOL measured by KIDSCREEN among adolescents with CP was conducted. On the other hand, they did not find a predictive value for motor impairment. This divergence is explained by the difference in target populations and measurement tools. A recent study carried out in Bangladesh using the CP-QOL Teens, published results similar to the study in hand (Power et al. 2020). The multiplicity of factors associated with QOL reported by this study and in the literature, requires us to be vigilant in interpreting the results.

Language disorders were a predictive factor for a lowered QOL. These results have been reported in the literature by several studies (Dobhal et al. 2014; Mezgebe et al. 2015; Power et al. 2020). They highlight the value of speech therapy, augmentative and alternative communication strategies and tools in improving QOL. A future study with this focus might highlight the human rights of children with CP when using assistive devices and technology for communication.

Consistent with the results of the studies of the SPARCLE project (Arnaud et al. 2008; Dickinson et al. 2007), a significant negative association between the intensity of pain and the QOL in child CP was found. It was among the predictors of QOL. In fact, the more intense the pain was, the more the QOL was altered. These results are consistent with the literature (Fairhurst et al. 2019; Mckinnon et al. 2019; Parkinson et al. 2013; Penner et al. 2013; Radsel, Osredkar & Neubauer 2016; Ramstad et al. 2011). Some authors using different measuring instruments from ours came to the same conclusions (Cristina et al. 2017; Schmidt et al. 2006). Indeed, it was shown through research published in 2016 that the presence of pain and the age of the child with CP explained approximately 14% of the variation in QOL (Findlay et al. 2016). The negative impact of pain on participation was explained by the fact that the discomfort caused by pain increases the rate of school absenteeism and results in restriction of participation in daily activities and family activities, leading children with CP to spend more days in bed (Houlihan et al. 2004).

Analyses showed that the higher the level of motor impairment according to the GMFCS, the lower the CP-QOL total. This joins several studies on the subject (Khare & Prajapati 2013; Pashmdarfard et al. 2017; Puspitasari, Rusmil & Gurnida 2013). According to the literature, the greater the motor impairment of these children, the more their autonomy in carrying out activities of daily living is limited, which largely explains poor QOL for physical health (Badia et al. 2014). This motor disability can lead to physical discomfort and difficulty in establishing relationships with other children (Arnaud et al. 2008). In addition, motor disability exposes them to restriction of their participation as an active member of family, school and community (Kerr, Mcdowell & Mcdonough 2007; Mei et al. 2014). Indeed, it was shown that their participation in physical and leisure activities is significantly reduced compared with children with normal development (Carlon et al. 2012).

This study highlights the benefit of a comprehensive assessment of children with CP, their family and environment in order to construct accurate estimates of their QOL. These findings also help stratify factors that could have an impact on QOL and subsequently helps in developing levels of intervention. In addition, the study in hand is based on the integration of the theoretical model of the ICF that offers a more exhaustive understanding of the experience of the child with CP. This study’s sample is heterogeneous because the authors were interested in all the clinical forms of the disease providing a realistic vision of this condition. The results from the study in hand are consistent with the relevant literature, which contributes to a greater external validity. In addition, the use of the CP QOL-Child, a specific validated measurement instrument for the QOL of children with CP, increases the credibility of the results. For medical practice, this study highlights the imperative of adopting a multidimensional approach, taking into account all bio-psycho-social aspects in the treatment of children with CP.

However, this study has some limitations. The limited sample does not allow for generalisation to the entire population of children with CP because only children who benefited from adequate care in a specialised centre were included, which is not always the case in Tunisian children with CP. This research is based on a transversal quantitative approach that can only paint a static picture of the experience of children with CP. Therefore, a longitudinal layout that follows the dynamic and evolving nature of QOL of these children and the various factors involved is better suited for this assessment. At the same time, the quantitative measurement of QOL alone does not fully describe the complexity of the experience of children with CP.

## Conclusion

Children with CP experience QOL alteration affecting every domain. Therefore, intervention with children with CP must be aware of their experience and rely on a thorough assessment of their QOL that is adapted to their family setting and cultural context. The multiplicity of the factors associated with QOL revealed by this study incites clinicians to adopt the ICF approach by displaying its practical implications on the efficiency of the medical intervention. Five out of the six predictive factors of QOL are modifiable through a multidisciplinary approach within the framework of the International Classification of Functioning, Disability and Health classification of functioning (ICF). Namely, swallowing and language disorders can be treated with dedicated orthophonic sessions and specific interventions, the level of motor impairment and the intensity of chronic pain can be improved through a global approach including multiple medical and non-medical interventions.
